# Rannasangpei Is a Therapeutic Agent in the Treatment of Vascular Dementia

**DOI:** 10.1155/2016/2530105

**Published:** 2016-05-17

**Authors:** Peng Wu, Yuandai Luo, Lifang Zhen, Xianda Hu, Ying Shang, Yinuo Liao, Huiyuan Xue, Fukai Huang, Wei Xiao

**Affiliations:** ^1^Southern Medical University, Guangzhou 510515, China; ^2^Beijing Tibetan Hospital, China Tibetology Research Center, Beijing 100029, China; ^3^Beijing Mentougou Hospital of Traditional Chinese Medicine, Beijing 102300, China

## Abstract

Rannasangpei (RSNP) is used as a therapeutic agent in the treatment of cardiovascular diseases, neurological disorders, and neurodegeneration in China; however, its potential use in the treatment of vascular dementia (VD) was unclear. In this study, our aim was to examine the neuroprotective effect of RSNP in a VD rat model, which was induced by permanent bilateral common carotid artery occlusion (2VO). Four-week administration with two doses of RSNP was investigated in our study. Severe cognitive deficit in the VD model, which was confirmed in Morris water maze (MWM) test, was significantly restored by the administration of RSNP. ELISA revealed that the treatments with both doses of RSNP could reinstate the cholinergic activity in the VD animals by elevating the production of choline acetyltransferase (ChAT) and reducing the acetylcholinesterase (AChE); the treatment of RSNP could also reboot the level of superoxide dismutase (SOD) and decrease malondialdehyde (MDA). Moreover, Western blot and quantitative PCR (Q-PCR) results indicated that the RSNP could suppress the apoptosis in the hippocampus of the VD animals by increasing the expression ratio of B-cell lymphoma-2 (Bcl-2) to Bcl-2-associated X protein (Bax). These results suggested that RSNP might be a therapeutic agent in the treatment of vascular dementia in the future.

## 1. Introduction

Vascular dementia (VD), which is caused by chronic cerebral hypoperfusion (CCH), hampers the intelligence of an individual, leading to the development of cognitive dysfunction syndrome [1, 2]. Epidemiological studies have shown that the prevalence of VD is remarkably high—VD is diagnosed in 20–30% of the total number of cases with dementia—in the elderly population of China and other countries of the world. Patients with VD are serious economic burdens to their families and the society [3, 4]. Several evidences have indicated that CCH aggravates cerebrovascular pathological changes [5, 6], such as central cholinergic dysfunction, oxidative injury, and apoptosis in hippocampus [7]. It is a well-known fact that central cholinergic neurotransmission is closely associated with learning and memory in humans [8]; reactive oxygen species cause oxidative stress that activates various signal transduction pathways involved in apoptosis. As a result, many cognitive deficits are caused in individuals [9]. All the above situations can eventually exacerbate the process of VD. However, the pathogenesis of VD is not fully understood till date. Owing to the complicated etiology and pathology of VD, it is difficult to develop a single-target therapy for patients with VD.

Rannasangpei (RSNP) (it is also known as* Ratna Samphel* or* 70 Flavors Pearl Pill*), a traditional Tibetan medicine [10], acts as a multitarget therapeutic agent in the treatment of cerebrovascular diseases. It has enormous therapeutic efficacy as it contains many ingredients, which are obtained from herbal, nonherbal, and mineral sources [11, 12]. RSNP originated from the classical prescription of* Ershiwuwei Zhenzhu* (*25 Flavors Pearl Pill*), which was recorded in the Tibetan medical masterpiece “Four Medical Tantra” in the 8th century. Thereafter, the classical prescription was improved and perfected by many generations of Tibetan physicians. The prescription used today was developed by Surkhar Nyamnyi Dorjé, a representative of the Southern School of Tibetan Medicine, in the mid-15th century. This Tibetan prescription mentions 70 different ingredients, including six superlative medicines [13], three myrobalan fruits [14], bellflower (*Codonopsis pilosula*), sandalwood (*lignum santali albi*), heartwood of rosewood (*lignun dalbergiae odoriferae*), bezoar (*calculus bovis*), and musk (*moschus*). RSNP also contains* Tsothel*—the king of essence [15].* Tsothel* is a form of detoxified mercury, which is carefully prepared through a series of standard detoxifying processes. Calcinated powder, which is prepared from precious stones and metals, such as pearl, turquoise, coral, nine-eye stone, gold, silver, copper, and iron, is also included in RSNP [16]. Because RSNP is a confidential Tibetan prescription, its precise composition and preparation method have still been kept as a secret (see Pharmacopoeia of the People's Republic of China for details [17]).

For hundreds of years, RSNP has been widely used in the treatment of various acute or chronic cerebrovascular and neuropsychological diseases, such as hypertension, stroke, cerebral hemorrhage, cerebral embolism, sequelae of traumatic brain injury, cerebral concussion, epilepsy, and cognitive impairment [18]. In particular, there was significant improvement in cognitive symptoms and daily living of patients with Alzheimer's disease when they were treated with RSNP at a high altitude [12]. To understand its therapeutic mechanisms, various preclinical studies have been conducted. Previous studies showed that RSNP can suppress the occlusion of middle cerebral artery, which induces neurologic deficits; it exerts neuroprotective effects by increasing superoxide dismutase (SOD), reducing lipid peroxidation, and regulating neuron-specific enolase and S-100*β* protein levels [19, 20]. Furthermore, animal experiments have proved that RSNP improves Alzheimer's disease by either preventing or degrading the aggregation of beta-amyloid, which reduces the formation of senile plaque [21, 22]. However, no previous research study has reported about its therapeutic effect on patients with VD. Therefore, the therapeutic mechanism through which RSNP improves the condition of patients with VD is unclear.

To determine the therapeutic effect of RSNP on patients with VD, we used a VD model in rat, which was induced by permanent, bilateral common carotid artery occlusion (2VO) [23]. We also included the ginkgo biloba extract (EGB) as a positive control; it contains ginkgo flavone glycosides, ginkgolides, and bilobalide. EGB is a well-known free radical scavenger and antioxidant drug. It can increase cerebral blood flow, prevent thrombosis, improve brain functions, and enhance memory [24, 25]. We evaluated whether RSNP improved the cognitive functions of VD in the animal model. Thereafter, we further investigated its therapeutic mechanisms by determining factors, such as the alteration of central cholinergic system, oxidative stress, cell apoptosis, and the related molecular regulations.

## 2. Materials and Methods

### 2.1. Drugs and Preparation

RSNP was purchased from a medicine pharmaceutical company named JinHei Tibetan (Qinghai, China). In this study, RSNP solution was freshly prepared for oral administration. Pills of RSNP were first crushed into a fine powder using mortar and pestle and then dissolved in distilled water at final concentrations of 14.09 and 56.35 mg/mL in separate patches. To dissolve the powder in distilled water, we stirred the suspensions at 300 rpm on a magnetic stirrer (Tianjin Wei Yi Technology Development Company, Tianjin, China) at room temperature till uniform suspensions were produced. The injection volume was 5 mL/kg. EGB was provided by Dr. Willmar Schwabe Gmbh & Co. Kg (Karlsruhe, Germany), which was also dissolved in distilled water (3.41 mg/mL) for further use. The preparation of EGB was the same as that of RSNP. All the other chemicals and materials were obtained from Beijing Tibetan Hospital, which is affiliated to the China Tibetology Research Center, Beijing, China.

### 2.2. Animal Models and Experimental Study Design

We obtained healthy male Sprague-Dawley rats (aged 3 months, weighing 220 ± 20 grams) from Si Beifu Laboratory Animal Science and Technology Co., Ltd. (Beijing, China). The rats were placed in cages; each cage contained a group of five rats. The cages were maintained at room temperature (23 ± 2°C); a relative humidity of 55 ± 5% was maintained in these cages; these rats were subjected to a light-dark cycle of 12 h (light from 7 a.m.–7 p.m.). These rats were provided* ad libitum* access to food and water throughout the study. The experimental protocols were approved by the Ethics Committee of Beijing Tibetan Hospital, China Tibetology Research Center. The animal experiments were performed according to the regulations of laboratory animal management, which were promulgated by the Ministry of Science and Technology of the People's Republic of China [1988] number 134. These regulations are in accordance with the internationally recognized guidelines of National Institute of Health (NIH), New York City, NY, USA.

In this study, 50 rats were randomly divided into five groups: (1) sham-operated group; (2) 2VO-induced VD model group; (3) RSNP-L (low dose) + 2VO group; (4) RSNP-M (medium dose) + 2VO group; (5) EGB + 2VO group. The rats were kept in cages for a week before the 2VO or sham surgeries. Five days after the surgery, the animals were orally given a definite volume of indicated drugs (5 mL/kg) or distilled water (5 mL/kg) once every day for four weeks; the sham-operated group and the VD model group were given same volume of distilled water (5 mL/kg). The administered doses of RSNP were 70.44 mg/kg (low dose) and 281.76 mg/kg (medium dose); EGB was administered at a dosage of 17.03 mg/kg.

### 2.3. Preparation of VD Model

The VD model of rats was produced by the bilateral common carotid arteries occlusion (2VO) method [26]. In brief, rats were anesthetized with choral hydrate at a dosage of 350 mg/kg. Throughout the surgery, the rectal temperature was maintained at 37 ± 0.5°C by placing a heating pad underneath the rat. The bilateral common carotid arteries were exposed by incising the ventral midline neck. Then, these carotid arteries were gently separated from the attached muscles, adjacent blood vessels, and nerves using glass dissecting tools. The bilateral common carotid arteries were double ligated with silk suture; a cut was made between the ligations to ensure a permanent ligature. At the end of the surgery, both the muscle layer and the skin layer were closed. After the surgery, the wound was disinfected with iodophil for seven days. In the sham-operated animals, none of the arteries were ligated.

### 2.4. Learning and Memory Assessment

For determining the spatial learning and memory capability, we carried out Morris water maze (MWM) test on the animals, after completing the four-week drug administration [26]. The experiment was performed in a circular water pool (150 cm in diameter), which was filled with opaque water (water was mixed with black ink) at 23 ± 1°C till a depth of 30 cm. The pool was equally divided into 4 quadrants, and an escape platform (10 cm × 10 cm) was placed at the center of one quadrant; the escape platform was submerged 2 cm under the surface of water. A video camera, which was connected to a computer, was placed above the pool to record and track the swimming paths of rats for subsequent analysis. During the entire experiment, same spatial signs and decorations were maintained around the maze.

During the first five consecutive days, each animal was trained four times per day to find the hidden platform. Every time, they were placed in the maze at the different starting points of different quadrants. All the animals followed the same sequence of starting positions. During each trial, each animal was given 90 s to find and mount on the hidden platform; they were allowed to rest on the platform for 30 s. If the animal did not find the platform within 90 s, it was gently guided onto the platform by the observer. Then, the animal was allowed to rest on the platform for 30 s. In each trial, the escape latency, which was defined as the amount of time spent by the animal in locating and mounting on the platform, was recorded and analyzed using the video analysis system, EthoVision (Noldus Information Technology, Wageningen, The Netherlands). These trials were called navigation tests. On the sixth day, a probe test was performed. In this test, the platform was removed to assess the spatial learning and memory capability of these animals. The percentage of the swimming time and the distance covered in the target quadrant, where the platform was originally placed, were recorded and analyzed.

### 2.5. Determination of Central Cholinergic Neurotransmission and Oxidative Stress Using Biochemical Analysis

The cholinergic neurotransmission was determined by detecting the activities of choline acetyltransferase (ChAT) and acetylcholinesterase (AChE); the oxidative stress in the brain was assessed by measuring the activity of superoxide dismutase (SOD) and the level of malondialdehyde (MDA). After the MWM experiment, the animals were sacrificed by overdosed choral hydrate (0.35 g/kg). The brain was immediately removed and placed on an ice-cold sterilized surface. Then, the hippocampi from both sides of the brain were dissected and weighed. The hippocampus of one side was stored at –80°C, and it was later used for performing quantitative polymerase chain reaction (Q-PCR). The tissues of the hippocampus from the other side were homogenized at 4°C with nine times its volume of 0.1 M phosphate buffer (pH = 7.4). About half of the homogenate was centrifuged at 4000 ×g for 15 min; the supernatant was collected for determining the amount of ChAT, AChE, SOD, and MDA, which was using ELISA kits according to the manufacturer's instructions (Beijing Cheng Lin Biological Technology Co., Beijing, China). The remaining homogenate of hippocampus was stored at –80°C for performing Western blot analysis subsequently.

### 2.6. Detection of Apoptotic Protein Expression Using Western Blotting

The expression of apoptotic regulatory protein, B-cell lymphoma-2 (Bcl-2), as well as a decrease in the level of Bcl-2-associated X protein (Bax), was assessed by both Western blotting and Q-PCR. The remaining homogenate was centrifuged at 1600 ×g for 10 min at 4°C; the supernatant was collected and the total supernatant protein was determined by Bradford assay. Samples containing 40 *μ*g of protein were denaturalized and separated on 10% sodium dodecyl sulfate polyacrylamide gel electrophoresis (SDS-PAGE) gels by electrophoresis. Then, the separated proteins were transferred onto nitrocellulose filter membranes. In Western blotting, the membranes were blocked for one hour with tris-buffered saline (TBS) containing 10% skimmed milk powder and 0.05% of Tween-20. Then, they were incubated for 2 h at 4°C with a primary antibody, which was diluted to 1 : 1000 in the blocking solution. In this experiment, the following primary antibodies were used: Bcl-2 antibody (mouse anti-rat, Novus Biologicals, USA), Bax antibody (mouse anti-rat, Abcam, Cambridge, UK), or *β*-actin antibody (rabbit anti-rat, Shanghai Kang Bioengineering Limited Company, Shanghai, China). The membranes were washed thrice with TBS containing 0.05% Tween-20 before they were incubated for 1.5 h at room temperature with a secondary antibody diluted to 1 : 5000 in the blocking solution. In this experiment, we used goat anti-rabbit IgG (Southern Biotech Associates, Birmingham, USA) as the secondary antibody. The relative intensity of the detected protein was quantified by gel image analysis system (Shanghai Day Technology Co., Shanghai, China).

### 2.7. Detection of Apoptotic mRNA Expression Using Real-Time Q-PCR

To determine the mRNA levels of Bcl-2, Bax, and *β*-actin n the hippocampus, real-time Q-PCR was performed. Total RNA was extracted from the hippocampus tissue using TRIzol reagent (Invitrogen, Carlsbad, USA). The purity as well as quantity of total RNA was detected by performing UV spectrophotometry using Biophotometer (Eppendorf, Hamburg, Germany). The integrity of the RNA was examined by performing agarose gels electrophoresis and visualized by an imaging system (Shanghai Day Technology Co., Shanghai, China). The qualified total RNA samples were treated with DNase I, and they were reverse transcribed into cDNA using Oligo(dT)15 primer (Promega, Madison, USA) and M-MLV Reverse Transcriptase (Promega, Madison, USA) according to the manufacturer's instruction. Quantitative Q-PCR was performed using synthesized cDNA, specific primers of interested genes (Table 1), and 2x SYBR Green PCR Master Mix (TOYOBO, Osaka, Japan) under the following reaction conditions: 1 cycle of 95°C for 10 min, 40 cycles of 95°C for 15 s, 60°C for 15 s, and 72°C for 30 s (ABI PRISM® 7500 Sequence Detection System, Applied Biosystems, Foster City, USA). Each amplification was performed in triplet. After amplification, the melting curve was used to verify the specificity of PCR products. *β*-actin was considered as an internal reference for normalization. Thus, we analyzed the relative mRNA level of Bcl-2 or Bax using the comparative cycle threshold method [27].

### 2.8. Statistical Analysis

Data were presented as mean ± SD. A two-way repeat measures analysis of variance (ANOVA) was applied to analyze the escape latencies of different groups in MWM test over a period of five days. Other data were analyzed using one-way ANOVA followed by the least significant difference (LSD) test. All the statistical analyses were performed using SPSS version 20.0 (IBM, Armonk, NY, USA). The results were assumed to be statistically significant when *P* < 0.05.

## 3. Results

### 3.1. RSNP Improved the Spatial Learning and Memory Deficits Induced by 2VO

The MWM test was used to observe the effect of RSNP on the spatial learning and memory capability of VD rat model, which was induced by 2VO.

**Figure 1:**
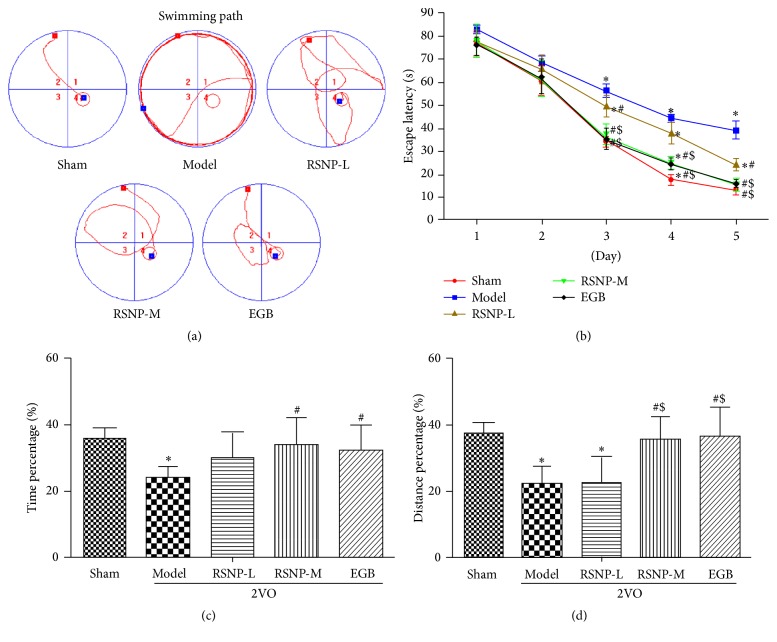
RSNP improves the impaired spatial learning and memory capability in VD animal model, which is proved by MWM test. (a) Representative pathways on the fifth day of navigation test, red block: start position, blue block: platform position; (b) escape latencies of rats in each group; (c) percentage of the time spent in the target quadrant in the probe test; (d) percentage of distance in the target quadrant in the probe test. Values were expressed as mean ± SD. Sham or sham-operated: sham-operated group; model: 2VO induced VD model group; RSNP-L: the group of VD model, which was administered with 70.44 mg/kg of RSNP for four weeks after the surgery; RSNP-M: the group of VD model, which was administered with 281.76 mg/kg of RSNP for four weeks after the surgery; EGB: the group of VD model, which was administered with 17.03 mg/kg of EGB for four weeks after the surgery; *n* = 10 per group; *∗*: compared to the sham-operated group, *P* < 0.05; #: compared to the model group, *P* < 0.05; $: compared to the RSNP-L group, *P* < 0.05.

As shown in Figure 1(b), the result of escape latency in the navigation test indicated a significant decrease in the escape latency with the passage of time (*F* = 924.053, *P* < 0.05). This indicates that the animals were indeed learning to memorize the position of the platform. There was statistically significant difference among the groups (*F* = 6.467, *P* < 0.05); the statistically significant difference among the groups depended on the day on which the trial was carried out during the five days of navigation test (*F* = 75.489, *P* < 0.05). Post hoc analysis showed that there was no significant difference in the escape latency of different groups in the first two days (*P* > 0.05); however, significant difference was detected from day 3 till day 5 of the navigation test (*P* < 0.05). Compared with the sham-operated group, the model group demonstrated significantly higher escape latency in the last 3 days (*P* < 0.05). This indicates that a clear learning disability was developed in the VD model group. Compared with the VD model group, both the RSNP-M treatment group, which received RSNP at a medium dose (281.76 mg/kg), and the EGB treatment group had a significantly lower escape latency of VD animals on day 3 to day 5 (*P* < 0.05), while the group that received RSNP at a low dose (70.44 mg/kg) (RSNP-L) also showed lower escape latency of VD animals on day 3 and day 5 (*P* < 0.05). Owing to the difference between the two doses of RSNP, the RSNP-M group spent significantly less time in finding the platform than the RSNP-L group on day 3 to day 5 (*P* < 0.05); there was no significant difference between RSNP-M and EGB groups (*P* > 0.05). These results indicated that both doses of RSNP improved the learning disability of VD animals, which was induced by 2VO; the medium dose of RSNP probably showed a better effect than the low dosage, and there was no significant difference in the recovery of learning ability of VD animals in RSNP-M group and EGB group.

The probe test was performed one day after the navigation test. The platform was removed before conducting the probe test. The percentage of swimming time and the distance of the target quadrant were used as indicators of memory capacity (Figures 1(c) and 1(d)). Compared to the sham-operated group, the VD model group spent considerably lesser time and covered lesser distance in the target quadrant (*P* < 0.05). This indicates that a memory deficit was induced by 2VO. In the RSNP-M and EGB groups, the VD animals' focus on the target quadrant was significantly better than those of the model group (*P* < 0.05); however, the memory capacity of RSNP-L group was not better than that of model group. Compared with the RSNP-L group, RSNP-M and EGB groups covered a significantly greater distance in the target quadrant (*P* < 0.05). Moreover, there was no significant difference between RSNP-M and EGB groups (*P* > 0.05). The results indicate that only a medium dose of RSNP alleviated the spatial memory deficit, which was previously induced in the experimental animals by 2VO.

In summary, RSNP treatment significantly improved the impaired learning and memory in the VD animal model. Furthermore, medium doses of RSNP definitely brought about a better improvement.

### 3.2. Effect of RSNP on the Cholinergic Activity of the Hippocampus of VD Animals

**Figure 2:**
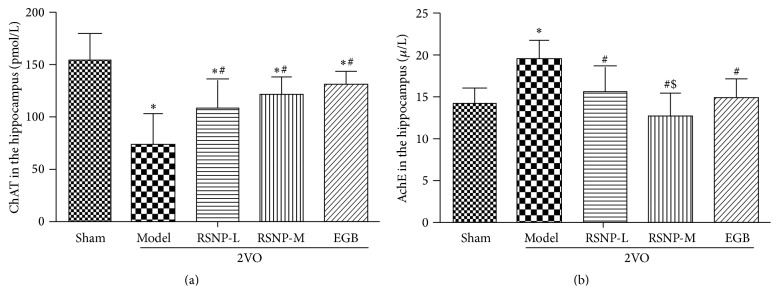
Effect of RSNP on the levels of ChAT (a), AChE (b) in the hippocampus of 2VO-induced VD rats. All the data were shown as mean ± SD, *n* = 10 per group. ^*∗*^ *P* < 0.05, compared with the sham-operated group; ^#^ *P* < 0.05, compared with the model group; ^$^ *P* < 0.05, compared with the RSNP-L group.

Since cholinergic neurotransmission was closely associated with the ability of learning and memory, we wanted to determine whether RSNP improved the learning and memory deficits in the VD animals by altering the cholinergic activities in the hippocampus. Therefore, we measured the levels of ChAT and AChE in the hippocampus of VD animals. The results (Figure 2) indicate that, in the model group, the level of ChAT was significantly lower than that in the sham-operated control group (*P* < 0.05). After being treated with either RSNP (70.44 or 281.76 mg/kg) or EGB, the level of ChAT in the treatment groups was significantly higher than that in the nontreated model group (*P* < 0.05). However, the level of ChAT was not completely restored to normal levels in the control group (*P* < 0.05).

Inversely, the content of AChE in the VD model group was significantly higher than that in the control group (*P* < 0.05); however, the AChE activity clearly decreased to normal levels in VD animals when they were treated with either RSNP or EGB (*P* < 0.05). Compared with the RSNP-L group, the RSNP-M treatment group showed a better recovery (*P* < 0.05). Furthermore, there was no significant difference between RSNP-M and EGB groups (*P* > 0.05).

These results indicate that there is a correlation between learning and memory capacity and cholinergic activity in the hippocampus. Both low and medium doses of RSNP could restore the altered cholinergic activity to normal levels in VD animals; however, the VD animals treated with the medium dose of RSNP showed better improvement in cholinergic activity.

### 3.3. Effect of RSNP on the Oxidative Stress Induced by 2VO in the Hippocampus

**Figure 3:**
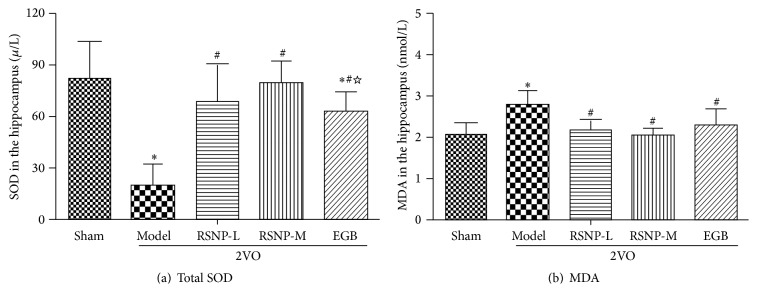
Effect of RSNP on the abnormal activity of SOD (a) and MDA (b) in the hippocampus of 2VO-induced VD rats. All data were shown as mean ± SD, *n* = 10 per group. ^*∗*^ *P* < 0.05, compared to the sham-operated group; ^#^ *P* < 0.05, compared with the VD model group; ^☆^ *P* < 0.05, compared with the RSNP-M group.

We also wanted to determine the effect of RSNP on oxidative stress, which plays a crucial role in damaging the brain of VD animals. For this purpose, we measured the levels of total SOD and MDA in the hippocampus (Figure 3). The results indicate that, in the VD model group, the total SOD was significantly lower than that in the sham-operated control group (*P* < 0.05). After being treated with either RSNP (70.44 or 281.76 mg/kg) or EGB, the activity of total SOD in VD animals increased sharply to become significantly higher than that in the model group (*P* < 0.05). Furthermore, in RSNP-M treatment group, the activity of SOD was even higher than that in EGB group (*P* < 0.05).

The content of MDA in the hippocampus of VD model group was significantly higher than that in the sham-operated control group (*P* < 0.05). Both doses of RSNP could effectively reduce the abnormally higher levels of MDA. Moreover, the abnormally higher MDA levels were also restored to normal levels in EGB group (*P* < 0.05). No significant difference was observed between RSNP and EGB groups (*P* > 0.05).

These results indicated that, in the VD animal model, RSNP could significantly decrease the oxidative stress induced by 2VO. Therefore, this Tibetan prescription has numerous beneficial effects on the brain.

### 3.4. Effects of RSNP on Apoptosis Regulatory Factors, Bcl-2 and Bax, and the Ratio of Bcl-2/Bax in the Hippocampus of VD Rat Model

**Figure 4:**
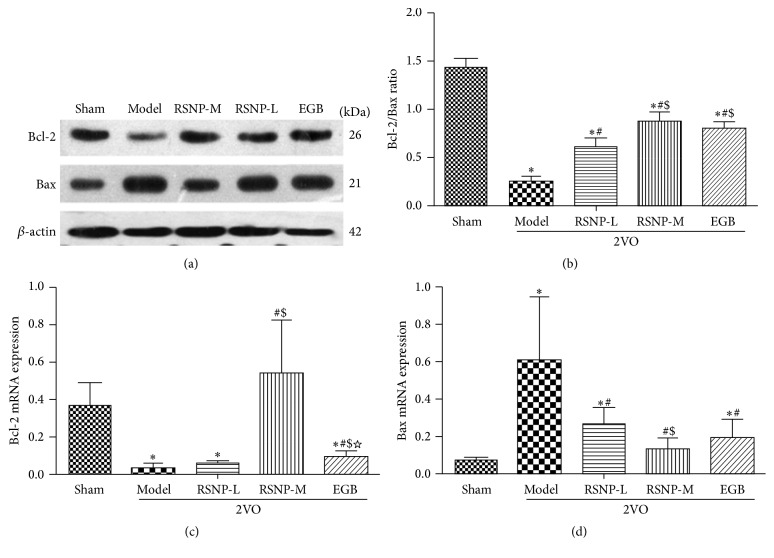
Effects of RSNP on the Bax and Bcl-2 levels in the hippocampus of VD model induced by 2VO. After completing the behavior test, the hippocampus was segregated and used to assess the expression of Bax and Bcl-2 by Western blot analysis (a). The intensities of Bcl-2 and Bax protein bands were used to determine the Bax/Bcl-2 ratio (b). The transcript levels of Bcl-2 (c) and Bax (d) genes in the hippocampus were also measured to verify the profile of protein expression. All the data were shown as mean ± SD, *n* = 10 per group. ^*∗*^ *P* < 0.05, compared with the sham-operated group; ^#^ *P* < 0.05, compared with the VD model group; ^$^ *P* < 0.05, compared with the RSNP-L group. ^☆^ *P* < 0.05, compared with the RSNP-M group.

To determine the effect of RSNP on the apoptosis regulatory factors, Bcl-2 and Bax, in this VD animal model, we used Western blotting analysis to detect the protein expression of Bcl-2 and Bax (Figure 4(a)). Then, a semiquantitative measurement was used to determine the ratio of Bcl-2/Bax (Figure 4(b)). The Western blotting results indicate that there was a decrease in the protein expression of Bcl-2, while the protein expression of Bax increased in the VD animal model. Furthermore, both RSNP (70.44 or 281.76 mg/kg) and EGB restored the protein expression of Bcl-2 and Bax to normal levels (the sham-operated control group showed normal levels of Bcl-2 and Bax) at different degrees. The result of semiquantitative analysis confirmed this observation: it showed a significant reduction in the Bcl-2/Bax ratio in VD model group (*P* < 0.05). Both RSNP and EGB could effectively prevent the 2VO-induced upregulation of Bax and the downregulation of Bcl-2. Therefore, the Bcl-2/Bax ratio was significantly increased (*P* < 0.05) in VD animals when they were treated with either RSNP or EGB. Compared with RSNP-L group, the RSNP-M and EGB groups had a higher Bcl-2/Bax ratio (*P* < 0.05). There was no significant difference between RSNP-M and EGB groups (*P* > 0.05).

To verify the protein expression data obtained by Western blotting, we performed real-time Q-PCR to examine the transcript levels of Bcl-2 and Bax (Figures 4(c) and 4(d)). The Q-PCR results indeed confirmed the protein expression of Bcl-2 and Bax. This indicates that when the VD animals were treated with RSNP (70.44 and 281.76 mg/kg) for four weeks, the abnormal transcript levels of Bcl-2 and Bax were improved in the hippocampus of VD rats; moreover, the medium dose of RSNP was more effective than the low dose of RSNP on both transcripts (*P* < 0.05). Interestingly, the RSNP-M group demonstrated a more protective effect than the EGB group, as indicated by the transcript level of Bcl-2 in the VD animals after the treatments (*P* < 0.05).

## 4. Discussion

In this study, we used the 2VO method to induce VD in experimental animals. This method was also used to prepare the model of chronic cerebral ischemia [28, 29] in which a persistent ischemia was developed in the animal's brain, causing cognitive dysfunction. Our current VD model showed serious impairment of learning and memory, which was caused by cerebral hypoperfusion [7, 30]. Therefore, this VD model was suitable for investigating the pharmacological mechanisms of RSNP, which has therapeutic effects on VD. After a 4-week oral administration of RSNP, the ability of learning and memory was improved greatly in the VD animal model. Further investigations proved that RSNP improved the cognitive dysfunction in VD animals by regulating cholinergic neurotransmission, reducing oxidative stress, and managing apoptotic regulators, such as Bax and Bcl-2, in the hippocampal area.

Although China is a multiethnic country, most people are of the Han Chinese race. In China, there are two major alternative systems of medicine: traditional Chinese medicine and ethnic medicine. Ethnic medicine has been quite effective in treating certain diseases. Traditional Tibetan medicine is one of the representative branches of ethnic medicine, and it has been practiced for many centuries [31]. RSNP, one of the most famous formulations of Tibetan medicine, is used to treat a wide range of diseases, such as hypertension, stroke, cerebral infarction, cerebral hemorrhage, and cardiomyopathy [18]. Previous research studies have reported that RSNP can be used to greatly reduce amnesia in patients with Alzheimer's disease [12, 32]. In this study, after administering RSNP orally for four weeks, the animals of VD model showed significantly lower escape latency in the navigation test. Moreover, in the probe test, their swimming time and distance in the target quadrant were also significantly lower. These results indicate that RSNP is efficient in improving the dysfunction of spatial learning and memory.

Central cholinergic system plays an important role in the spatial learning and memory. Acetylcholine (ACh), the actual neurotransmitter, is synthesized from a reaction between choline and acetyl coenzyme A; this reaction is catalyzed by ChAT in cholinergic synapses. After ACh is released into synaptic cleft, AChE rapidly degrades it to avoid excessive cholinergic neurotransmission. Therefore, ChAT and AChE actively control the homeostasis of cholinergic system [33]. In patients with cerebral ischemia, the lesions are located in the hippocampus and cortex where the activity of ChAT increases, while the activity of AChE decreases. As a result, the synthesis of ACh is reduced, resulting in the dysfunction of cholinergic system. All these events cause the disability of learning and memory, which eventually leads to dementia in these patients [34]. In this study, both doses of RSNP were effective in reducing AChE and enhancing ChAT activity. This indicates that RSNP improves impaired learning and memory in VD animal model by increasing cholinergic neurotransmission.

Free radicals are found extensively in organisms. They cause damage to proteins and DNAs, triggering serious diseases [35–37]. There were many endogenous antioxidants in the human body, including SOD, catalase, and cytochrome. These antioxidants scavenge free radicals and maintain a dynamic equilibrium. However, oxidative stress is significant in patients with cerebral ischemia. Since the brain is rich in lipids, free radicals can easily stimulate lipid peroxidation [38], leading to the generation of many toxic free radicals; these free radicals cannot be scavenged by the endogenous antioxidants in a timely manner. Therefore, these excessive free radicals cause damage to proteins and DNAs, triggering an intense damage to the brain [39]. SOD, which was an important antioxidant enzyme in the body, can remove superoxide anion radicals and inhibit lipid peroxidation. Thus, it can protect cells from further damage [40]. On the other hand, MDA is a product of lipid peroxidation; its content in the brain indicates not only the level of lipid peroxidation but also the severity with which body cells are attacked by free radicals [28, 41]. In this study, we have measured the activity of SOD and the level of MDA in the hippocampus to estimate the antioxidant activity and the level of oxidative stress, respectively. RSNP efficiently recovered the pathologically reduced SOD and the increased MDA in VD animals induced with 2VO surgery. This indicates that RSNP is beneficial to the animals of VD model as it eliminates the excessive accumulation of free radicals, which are produced due to chronic cerebral ischemia.

Hippocampus, a crucial structure that governs learning ability and memory, is found to be closely associated with spatial cognitive function. Therefore, it is very vulnerable to apoptosis in patients inflicted with brain ischemia and hypoperfusion. Previous studies have reported that the Bcl-2 protein family plays a vital role in the regulation of apoptosis [42]. Bcl-2 and Bax are the two major factors influencing the regulation of apoptosis; Bcl-2 prevents the release of cytochrome C from mitochondria, and it also activates caspases to inhibit apoptosis. In contrast, Bax protein directly induces the release of cytochrome C from mitochondria into the cytoplasm, thereby promoting apoptosis [43]. To further explore the function of RSNP in inhibiting cell apoptosis, we performed Western blot and real-time Q-PCR. With these methods, we detected the expression levels of Bcl-2 and Bax both as protein and as mRNA. In the VD model group, the level of Bax increased significantly, while the level of Bcl-2 decreased significantly. After the administration of RSNP for four weeks, the level of Bcl-2 increased significantly, while the level of Bax decreased. Thus, the Bcl-2/Bax ratio increased in VD animals. This result indicated that RSNP reduces apoptosis in the brain by regulating the levels of Bcl-2 and Bax.

For many centuries, RSNP has been widely used in the treatment of chronic cerebrovascular diseases and neural system diseases, but the therapeutic mechanisms of RSNP have not yet been deciphered. Ours was the first study to elucidate the neuroprotective effects of RSNP in the animal model of VD. The protective mechanisms of RSNP probably involve the reduction of oxidative stress, which ultimately leads to a decrease in proapoptotic Bax/Bcl-2 ratio and the restoration of cholinergic neurotransmission. A medium dose of RSNP (281.76 mg/kg) provided neuroprotective effects, which were the same as those offered by EGB. This study proves that RSNP can be considered as a potential therapeutic agent in the treatment of VD.

## Figures and Tables

**Table 1:** Primer sequences used for Q-PCR.

Gene name	Primer sequences	Product length
Bcl-2-F	5′-GGGACGCGAAGTGCTATTGGT-3′	208 bp
Bcl-2-R	5′-CTCAGGCTGGAAGGAGAAGAT-3′	208 bp
Bax-F	5′-GTTTCATCCAGGATCGAGCAGA-3′	265 bp
Bax-R	5′-GCAAAGTAGAAGAGGGCAACCAC-3′	265 bp
*β*-actin-F	5′ AGGGAAATCGTGCGTGACAT	150 p
*β*-actin-R	5′ GAACCGCTCATTGCCGATAG	150 p
